# Analysis of genotype distribution of thalassemia and G6PD deficiency among Hakka population in Meizhou city of Guangdong Province

**DOI:** 10.1002/jcla.23140

**Published:** 2019-12-03

**Authors:** Heming Wu, Qiuyan Zhu, Hua Zhong, Zhikang Yu, Qunji Zhang, Qingyan Huang

**Affiliations:** ^1^ Center for Precision Medicine Meizhou People's Hospital (Huangtang Hospital) Meizhou Academy of Medical Sciences Meizhou Hospital Affiliated to Sun Yat‐sen University Meizhou China; ^2^ Guangdong Provincial Key Laboratory of Precision Medicine and Clinical Translational Research of Hakka Population Meizhou People's Hospital (Huangtang Hospital) Meizhou Academy of Medical Sciences Meizhou Hospital Affiliated to Sun Yat‐sen University Meizhou China; ^3^ Guangdong Provincial Engineering and Technology Research Center for Clinical Molecular Diagnostics and Antibody Therapeutics Meizhou China; ^4^ Meizhou Municipal Engineering and Technology Research Center for Molecular Diagnostics of Major Genetic Disorders Meizhou People's Hospital (Huangtang Hospital) Meizhou Academy of Medical Sciences Meizhou Hospital Affiliated to Sun Yat‐sen University Meizhou China

**Keywords:** G6PD, genotype distribution, Meizhou city, southern China, thalassemia

## Abstract

**Objective:**

The aim of the study was to explore genotype distribution thalassemia and G6PD deficiency in Meizhou city, China.

**Methods:**

A total of 16 158 individuals were involved in thalassemia genetic testing. A total of 605 subjects were screened for common Chinese *G6PD* mutations by gene chip analysis. Genotypes and allele frequencies were analyzed.

**Results:**

A total of 5463 cases carried thalassemia mutations were identified, including 3585 cases, 1701 cases, and 177 cases with α‐, β‐, and α + β‐thalassemia mutations, respectively. ‐‐^SEA^ (65.12%), ‐α^3.7^ (19.05%), and ‐α^4.2^ (8.05%) deletion were the main mutations of α‐thalassemia, while IVS‐II‐654(C → T) (40.39%), CD41‐42(‐TCTT) (32.72%), ‐28(A → G) (10.11%), and CD17(A → T) (9.32%) mutations were the principal mutations of β‐thalassemia in Meizhou. There were significant differences in allele frequencies in some counties. Genetic testing for G6PD deficiency, six mutation sites, and one polymorphism were detected in our study. A total of 198 alleles with the mutation were detected among 805 alleles (24.6%). G6PD Canton (c.1376 G → T) (45.96%), G6PD Kaiping (c.1388 G → A) (39.39%), and G6PD Gaohe (c.95 A → G) (9.09%) account for 94.44% mutations, followed by G6PD Chinese‐5 (c.1024 C → T) (4.04%), G6PD Viangchan (c.871G → A) (1.01%), and G6PD Maewo (c.1360 C → T) (0.51%). There were some differences of the distribution of *G6PD* mutations among eight counties in Meizhou.

**Conclusions:**

The ‐‐^SEA^, ‐α^3.7^, and ‐α^4.2^ deletion were the main mutations of α‐thalassemia, while IVS‐II‐654(C → T), CD41‐42(‐TCTT), ‐28(A → G), and CD17(A → T) mutations were the principal mutations of β‐thalassemia in Meizhou. *G6PD* c.1376 G → T, c.1388 G → A, and c.95 A → G were the main mutations of G6PD deficiency. There were some differences of the distribution of thalassemia and *G6PD* mutations among eight counties in Meizhou.

## 1 INTRODUCTION

Hemoglobin disease and glucose‐6‐phosphate dehydrogenase (G6PD) deficiency are widespread human erythrocytogenetic diseases that affect millions of population.^1^, ^2^ The geographical distribution of G6PD deficiency and thalassemia is closely related to the past and present epidemic of malaria, because it has the selective advantage of anti‐malaria infection.^3^, ^4^

Thalassemia is an inherited autosomal recessive disease with microcytic hypochromic anemia resulting from reduced or absent synthesis of one or more of the globin chains of hemoglobin. As one of the commonest monogenic disorder in the world, thalassemia assumes diversity in clinical phenotypes varying from almost asymptomatic to lethal hemolytic anemia.^5^, ^6^, ^7^ Thalassemia is the most common monogenic disorder in the world and is especially prevalent in Mediterranean countries, Southeast Asia, Africa, Middle East, and in the Indian subcontinent. There are two main types of thalassemia, α and β.^8^, ^9^ According to the reports of previous researches, thalassemia was mainly prevalent in the population of southern areas of the Yangtze River in southern China,^10^, ^11^, ^12^, ^13^ especially in three most southerly provinces of Guangxi,^14^, ^15^, ^16^ Guangdong,^17^ and Hainan,^18^, ^19^ in which individuals seems to be selective advantages to carry such mutations.

Hereditary G6PD deficiency is one of the most common genetic enzyme deficiency diseases in the world. G6PD deficiency is an X‐linked incomplete dominant inherited disease. The *G6PD* gene is located on chromosome Xq28 which consists of 13 exons and 12 introns, encoding 515 amino acids. The deficiency is widely distributed and occurs in about 400 million people worldwide.^20^ G6PD deficiency has an obvious geographical distribution in the mainland China, and it is higher in the provinces south of the Yangtze River, including Guangdong, Hainan, Guangxi, Yunnan, Guizhou, and Sichuan provinces.^21^, ^22^, ^23^

Meizhou is a city located in the northeast of Guangdong Province, and most of the residents living in this area are Hakka peoples. Hakka is an intriguing Han Chinese population that mainly inhabit in southern China who migrated to south originally from northern China.^24^ Meizhou city consists of eight counties including Wuhua, Fengshun, Dabu, Jiaoling, Meijiang, Meixian, Pingyuan, and Xingning, where custom, lifestyle, and diet manifest some distinctions in certain extent.

Population screening and genetic counseling are important to prevent the birth of children with thalassemia major. Using genetic analysis for prenatal diagnosis can diagnose thalassemia major fetuses in early pregnancy and terminate pregnancy in time, so as to avoid the birth of thalassemia major patients, which is an effective method to prevent this disease at present. Precise mutation frequencies studies in different populations will help healthcare programs to control thalassemia.^25^, ^26^ G6PD deficiency causes neonatal hyperbilirubinemia and chronic hemolytic anemia. Although most affected individuals are asymptomatic, exposure to oxidative stressors, such as certain drugs or infection, can elicit acute hemolysis.^27^, ^28^ Meizhou is regarded as underdeveloped and backward city in Guangdong Province. As a social medical and health problem, thalassemia has brought great challenges to the development of Meizhou region. Here, we perform a survey of thalassemia and G6PD deficiency to analyze the feature of genotypes distribution and frequencies among eight counties of Meizhou area.

## 2 MATERIALS AND METHODS

### 2.1 Subjects

**Figure 1:**
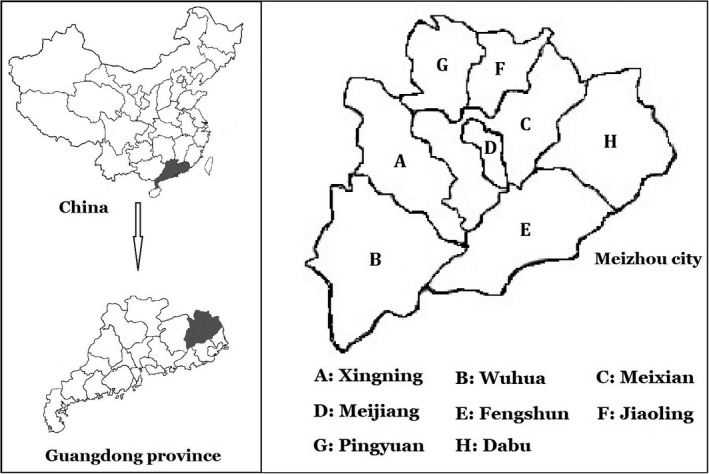
The geographical position of the eight counties in Meizhou

A total of 16 158 individuals who visited Meizhou People's Hospital (Huangtang Hospital) from January 2015 to May 2018 were involved in thalassemia genetic testing in this study. From February 2016 to May 2018, 605 subjects were screened for common Chinese G6PD mutations by gene chip analysis. The subjects included patients who went to cardiovascular disease center, prenatal diagnosis center, reproductive medicine center, physical examination center, pediatrics, gynecology, and other professional departments of our hospital, excluding patients with blood diseases. Figure 1 shows the location of the eight counties in Meizhou. This study was approved by the Ethics Committees of Meizhou People's Hospital (Huangtang Hospital), Meizhou Hospital Affiliated to Sun Yat‐sen University (Guangdong Province, China) and was conducted according to the Declaration of Helsinki for biomedical research involving human participants. All participants provided written informed consent before enrollment in the study.

### 2.2 Genetic testing for thalassemia

#### 2.2.1 Hematological studies and hemoglobin electrophoresis analysis

Samples were obtained via venipuncture of an antecubital vein, and then, 2 mL of peripheral blood was collected in EDTA anticoagulant tube. Sysmex XE‐2100 blood analyzer (Sysmex Corporation of Japan, Block scientific Inc based on New York) was used to determine erythrocyte correlative indices following the standard operating procedures. Hemoglobin electrophoresis analysis was performed by Sebia capillary electrophoresis system (Sebia, Inc) compiling with standard operating procedures. Subjects detected to low mean corpuscular volume (MCV) values (<82 fL) and (or) lower mean corpuscular hemoglobin (MCH) values (<27 pg) were thought as suspicious thalassemia carriers. Subjects with low HbA_2_ (<2.5%) were considered probable α‐thalassemia carriers, whereas with high HbA_2_ (>3.5%) were deemed possible β‐thalassemia carriers.

#### 2.2.2 Molecular analysis

Peripheral blood was collected with EDTA anticoagulant tube, in which genomic DNA was extracted from leukocytes. DNA concentration was quantified using NanoDrop 2000™ Spectrophotometer (Thermo Fisher Scientific). Gap‐polymerase chain reaction (gap‐PCR) and flow‐through hybridization technology (Hybribio Limited, Chaozhou, China) were used to detect α‐thalassemia mutations, both deletional mutations (‐‐^SEA^, ‐α^3.7^, and ‐α^4.2^) and non‐deletional mutations (Hb Constant Spring (α^CS^α) (CD142,TAA → CAA), Hb Quong Sze (α^QS^α) (CD125,CTG → CCG), and Hb Westmead (α^WS^α) (CD122,CAC → CAG)). Sixteen common non‐deletional mutations in β‐globin gene were detected by PCR and flow‐through hybridization technology (Hybribio Limited, China): CD41‐42(‐TCTT), CD43(G → T), IVS‐II‐654(C → T), CD17(A → T), CD14‐15(+G), ‐28(A → G), −29(A → G), CD71‐72(+A), CD26(G → A), IVS‐I‐1(G → T), IVS‐I‐1(G → A), CD27‐28(+C), IVS‐I‐5(G → C), Cap + 40‐43(‐AAAC), initiation codon (T → G), and CD31(‐C).

### 2.3 Genetic testing for G6PD deficiency

Genomic DNA was extracted from EDTA anticoagulant blood of subjects using the QIAamp DNA Blood Mini Kit (Qiagen). Amplification was performed using the *G6PD* Gene Typing Detection Kit (gene chip assay) (Sinochips Bioscience Co.). We detected the six mutation and one polymorphism sites in *G6PD* gene most commonly seen in the Chinese population by gene chip assay, including G6PD Gaohe (c.95 A → G, 32 His → Arg), G6PD Viangchan (c.871G → A, 291 Val → Met), G6PD Chinese‐5 (c.1024 C → T, 342 Leu → Phe), G6PD Maewo (c.1360 C → T, 454 Arg → Cys), G6PD Canton (c.1376 G → T, 459 Arg → Leu), G6PD Kaiping (c.1388 G → A, 463 Arg → His), and one polymorphism (c.1311 C → T, rs2230037).

### 2.4 Statistical analysis

This study used SPSS statistical software version 20.0 (International Business Machines Corporation) to analyze data, and the results would be displayed with corresponding proportion. Descriptive analysis and Pearson chi‐square test were used to compare the frequencies of genotype and allele among different counties in Meizhou region. *P* < .05 was considered to statistical difference.

## 3 RESULTS

A total of 16 158 blood samples were obtained and analyzed from eight counties of Meizhou area. A total of 8701 cases with microcytosis (MCV < 82 fL and/or MCH < 27 pg) were found, and corresponding percentages of microcytosis in Xingning, Wuhua, Meixian, Meijiang, Fengshun, Jiaoling, Pingyuan, and Dabu were 56.96% (1792/3146), 56.87% (1750/3077), 51.29% (1407/2743), 55.14% (1025/1859), 48.76% (1023/2098), 48.33% (652/1349), 55.54% (536/966), and 55.98% (515/920), respectively. The corresponding abnormal ratio of hemoglobin in Xingning, Wuhua, Meixian, Meijiang, Fengshun, Jiaoling, Pingyuan, and Dabu were 52.64% (1656/3146), 52.00% (1600/3077), 46.34% (1271/2743), 47.98% (892/1859), 47.47% (996/2098), 44.48% (600/1349), 48.96% (473/966), and 50.98% (469/920), respectively.

The result of the incidence rate of α‐ and β‐thalassemia of eight counties in Meizhou area is shown in Table 1. A total of 5463 cases of thalassemia were identified including 3585 cases of α‐thalassemia, 1701 cases of β‐thalassemia, and 177 cases of α‐compound β‐thalassemia. The total prevalence of thalassemia in Meijiang (31.84%, *P* = .043) was lower than other counties in Meizhou area. However, the relative proportions of α‐ and β‐thalassemia did not differ greatly among eight counties. The prevalence of α‐thalassemia in Meijiang (20.73%, *P* = .046) was lower than other counties in Meizhou area. The prevalence of Hb H disease in Wuhua (2.31%, *P* = .008) was higher than other counties in Meizhou area, while in Jiaoling (0.89%, *P* = .015) was lower than other counties.

**Table 1:** The prevalence of α‐ and β‐thalassemia in different counties of Meizhou area

Variable	Case number (n = 16 158) (percentage, %)								Total (n = 16 158)
	Xingning (n = 3146)	Wuhua (n = 3077)	Meixian (n = 2743)	Meijiang (n = 2098)	Fengshun (n = 1859)	Jiaoling (n = 1349)	Pingyuan (n = 966)	Dabu (n = 920)	
α‐thalassemia	693 (21.96)	703 (22.85)	615 (22.42)	435 (20.73)^a^	411 (22.11)	295 (21.87)	230 (23.81)	203 (22.07)	3585 (22.19)
α‐thalassemia silent	188 (5.98)	174 (5.65)	142 (5.18)	95 (4.53)	99 (5.33)	84 (6.23)	60 (6.21)	46 (5.00)	888 (5.50)
α‐thalassemia trait	454 (14.43)	458 (14.88)	423 (15.42)	316 (15.06)	277 (14.90)	199 (14.75)	150 (15.53)	139 (15.11)	2416 (14.95)
HB H disease	51 (1.62)	71 (2.31)^a^	50 (1.82)	24 (1.14)	35 (1.88)	12 (0.89)^a^	20 (2.07)	18 (1.96)	281 (1.74)
β‐thalassemia	338 (10.74)	336 (10.92)	275 (10.03)	218 (10.39)	184 (9.80)	144 (10.67)	111(11.49)	95 (10.33)	1701 (10.53)
β‐thalassemia trait	333 (10.58)	330 (10.72)	273 (9.95)	214 (10.20)	182 (9.79)	142 (10.53)	111 (11.49)	93 (10,11)	1678 (10.38)
β‐thalassemia major	5 (0.16)	6 (0.19)	2 (0.07)	4 (0.19)	2 (0.11)	2 (0.15)	0 (0)	2 (0.22)	23 (0.14)
α‐compound β‐thalassemia	40 (1.27)	40 (1.30)	29 (1.06)	15 (0.71)	20 (1.08)	12 (0.89)	7 (0.72)	14 (1.52)	177 (1.10)
Total	1071 (34.04)	1079 (35.07)	919 (33.50)	668 (31.84)^a^	615 (33.08)	451 (33.43)	348 (36.02)	312 (33.91)	5463 (33.81)

The shaded cells show that there are statistically significant differences compared with other counties.

a Manifests that compared with other counties, *P* < .05.

Allele frequencies of α‐ and β‐thalassemia of eight counties in Meizhou area are exhibited in Table 2. A total of 5952 mutant chromosomes including 4063 α‐thalassemia mutant chromosomes and 1889 β‐thalassemia mutant chromosomes were identified. Compared with other counties, Wuhua assumed higher allele frequencies in both α‐thalassemia (13.39%, *P* = .032) and ‐α^3.7^ deletion (2.75%, *P* = .045), and higher frequencies in α^WS^α allele of Xingning (0.60%, *P* = .001), in ‐α^4.2^ deletion of Jiaoling (1.45%, *P* = .023), in α^CS^α allele of Dabu (0.98%, *P* = .006), and in IVS‐II‐654(C → T) allele of Pingyuan (3.16%, *P* = .018) were showed compared with other counties separately. IVS‐II‐654(C → T) mutation was found dominance in most of these counties, while Meixian and Fengshun had a higher percentage in CD41‐42(‐TCTT) (1.99% and 2.27%, respectively) allele. The β‐thalassemia mutations in the order of allele frequency between eight regions of Meizhou, the mutations were IVS‐II‐654(C → T) > CD41‐42(‐TCTT) > ‐28(A → G) > CD17(A → T) in Xingning, Meijiang, Jiaoling and Pingyuan (similar to Jiangxi), IVS‐II‐654(C → T) > CD41‐42(‐TCTT) > CD17(A → T) > ‐28(A → G) in Wuhua and Dabu (similar to Fujian), CD41‐42(‐TCTT) > IVS‐II‐654(C → T) > CD17(A → T) > ‐28(A → G) in Meixian and Fengshun.

**Table 2:** Allele frequencies of α‐ and β‐thalassemia in different counties of Meizhou area

Mutant chromosomes	Allele number (percentage, %)								Total (n = 32 316)
	Xingning (n = 6292)	Wuhua (n = 6154)	Meixian (n = 5486)	Meijiang (n = 4196)	Fengshun (n = 3718)	Jiaoling (n = 2698)	Pingyuan (n = 1932)	Dabu (n = 1840)	
α‐thalassemia	790 (12.56)	824 (13.39)^a^	693 (12.63)	478 (11.39)	473 (12.72)	322 (11.93)	253 (13.10)	230 (12.50)	4063 (12.57)
(‐‐^SEA^)deletion	515 (8.18)	521 (8.47)	458 (8.34)	329 (7.84)	295 (7.93)	202 (7.49)	169 (8.75)	157 (8.53)	2646 (8.19)
(‐α^3.7^)deletion	154 (2.45)	169 (2.75)^a^	135 (2.46)	82 (1.95)	95 (2.56)	57 (2.11)	49 (2.53)	33 (1.79)	774 (2.40)
(‐α^4.2^)deletion	67 (1.06)	63 (1.02)	49 (0.89)	39 (0.93)	33 (0.89)	39 (1.45)^a^	22 (1.14)	15 (0.82)	327 (1.01)
α^CS^α(CD142,TAA → CAA)	13 (0.21)	31 (0.50)	38 (0.69)	18 (0.43)	25 (0.67)	13 (0.48)	5 (0.26)	18 (0.98)^a^	161 (0.50)
α^WS^α (CD122,CAC → CAG)	38 (0.60)^a^	29 (0.47)	10 (0.18)	9 (0.21)	15 (0.40)	6 (0.22)	8 (0.41)	5 (0.27)	120 (0.37)
α^QS^α (CD125,CTG → CCG)	3 (0.05)	11 (0.18)	3 (0.05)	1 (0.02)	9 (0.24)	5 (0.19)	0 (0)	2 (0.11)	34 (0.11)
(‐α^HK^)deletion	0 (0)	0 (0)	0 (0)	0 (0)	1 (0.03)	0 (0)	0 (0)	0 (0)	1 (0.01)
β‐thalassemia	382 (6.07)	380 (6.17)	305 (5.56)	237 (5.65)	201 (5.41)	156 (5.78)	119 (6.16)	109 (5.92)	1889 (5.85)
IVS‐II‐654(C → T)	157 (2.50)	144 (2.34)	108 (1.97)	97 (2.31)	75 (2.02)	75 (2.78)	61 (3.16)^a^	46 (2.50)	763 (2.36)
CD41‐42 (‐TCTT)	103 (1.64)	142 (2.31)	109 (1.99)	70 (1.67)	85 (2.27)	42 (1.56)	32 (1.66)	35 (1.90)	618 (1.91)
‐28 (A → G)	54 (0.86)	34 (0.55)	28 (0.51)	22 (0.52)	14 (0.38)	17 (0.63)	17 (0.88)	5 (0.27)	191 (0.59)
CD17 (A → T)	40 (0.64)	43 (0.70)	31 (0.57)	20 (0.48)	17 (0.46)	8 (0.30)	7 (0.36)	10 (0.54)	176 (0.54)
CD27‐28 (+C)	10 (0.16)	1 (0.02)	6 (0.11)	7 (0.17)	0 (0)	4 (0.15)	2 (0.10)	8 (0.43)	38 (0.12)
CD26 (G → A)	2 (0.03)	1 (0.02)	9 (0.16)	10 (0.24)	6 (0.16)	8 (0.30)	0 (0)	2 (0.11)	38 (0.12)
CD71‐72 (+A)	8 (0.13)	7 (0.11)	4 (0.07)	2 (0.05)	1 (0.03)	2 (0.07)	0 (0)	1 (0.05)	25 (0.08)
Cap+40‐43 (‐AAAC)	0 (0)	3 (0.05)	5 (0.09)	6 (0.14)	2 (0.05)	0 (0)	0 (0)	2 (0.11)	18 (0.06)
CD14‐15 (+G)	2 (0.03)	0 (0)	1 (0.02)	1 (0.02)	1 (0.03)	0 (0)	0 (0)	0 (0)	5 (0.02)
‐29 (A → G)	5 (0.08)	3 (0.05)	2 (0.04)	1 (0.02)	0 (0)	0 (0)	0 (0)	0 (0)	11 (0.03)
CD43 (G → T)	1 (0.02)	2 (0.03)	1 (0.02)	0 (0)	0 (0)	0 (0)	0 (0)	0 (0)	4 (0.01)
IVS‐I‐1	0 (0)	0 (0)	1 (0.02)	1 (0.02)	0 (0)	0 (0)	0 (0)	0 (0)	2 (0.01)

The shaded cells show that there are statistically significant differences compared with other counties.

a manifests that compared with other counties, *P* < .05.

The result of genotypes of different counties in Meizhou area is shown in Table 3, showing subtle differences among each county. There were higher genotype frequencies in ‐α^4.2^/αα, β^N^/β^N^ of Jiaoling (2.45%, *P* = .002), in ‐‐^SEA^/‐α^3.7^, β^N^/β^N^ (1.55%, *P* = .030) and αα/αα, β^IVS‐II‐654^/β^N^ (5.80%, *P* = .015) of Pingyuan, in α^CS^α/αα, β^N^/β^N^ of Meixian (0.95%, *P* = .010), and in ‐‐^SEA^/α^CS^α, β^N^/β^N^ of Dabu (1.20%, *P* < .001) compared with other counties. There are lower frequencies in α^CS^α/αα, β^N^/β^N^ of Xingning (0.25%, *P* = .006) and in αα/αα, β^‐28^/β^N^ of Dabu (0.22%, *P* = .019) compared with other counties.

**Table 3:** The mutant genotypes of different counties in Meizhou area

Mutant genotype	Case number (n = 16 158) (percentage, %)								Total (n = 16 158)
	Xingning (n = 3146)	Wuhua (n = 3077)	Meixian (n = 2743)	Meijiang (n = 2098)	Fengshun (n = 1859)	Jiaoling (n = 1349)	Pingyuan (n = 966)	Dabu (n = 920)	
α‐thalassemia	693 (22.03)	703 (22.85)	615 (22.42)	435 (20.73)^a^	411 (22.11)	295 (21.87)	230 (23.81)	203 (22.07)	3585 (22.19)
‐‐^SEA^/αα, β^N^/β^N^	435 (13.83)	425 (13.81)	394 (14.36)	298 (14.20)	248 (13.34)	181 (13.42)	146 (15.11)	129 (14.02)	2256 (13.96)
‐α^3.7^/αα, β^N^/β^N^	115 (3.66)	114 (4.68)	99 (3.61)	60 (2.86)	68 (3.66)	47 (3.48)	34 (3.52)	28 (3.04)	565 (3.42)
‐α^4.2^/αα, β^N^/β^N^	46 (1.46)	42 (1.36)	34 (1.24)	28 (1.33)	18 (0.97)	33 (2.45)^a^	18 (1.86)	14 (1.52)	233 (1.44)
‐‐^SEA^/‐α^3.7^, β^N^/β^N^	26 (0.83)	37 (1.20)	21 (0.77)	16 (0.76)	15 (0.81)	6 (0.44)	15 (1.55)^a^	5 (0.54)	141 (0.87)
α^CS^α/αα, β^N^/β^N^	8 (0.25)^a^	17 (0.55)	26 (0.95)^a^	14 (0.67)	14 (0.75)	11 (0.82)	4 (0.41)	7 (0.76)	101 (0.62)
α^WS^α/αα, β^N^/β^N^	27 (0.86)	18 (0.58)	9 (0.33)	7 (0.33)	13 (0.70)	4 (0.30)	8 (0.83)	4 (0.43)	90 (0.56)
‐‐^SEA^/‐α^4.2^, β^N^/β^N^	17 (0.54)	11 (0.36)	14 (0.51)	6 (0.29)	10 (0.54)	4 (0.30)	4 (0.41)	1 (0.10)	67 (0.41)
‐‐^SEA^/α^CS^α, β^N^/β^N^	4 (0.13)	14 (0.45)	11 (0.40)	2 (0.10)	7 (0.38)	2 (0.15)	1 (0.10)	11 (1.20)^a^	52 (0.32)
α^QS^α/αα, β^N^/β^N^	3 (0.10)	7 (0.23)	2 (0.07)	1 (0.05)	9 (0.48)	5 (0.37)	0 (0)	2 (0.22)	29 (0.18)
‐‐^SEA^/α^WS^α, β^N^/β^N^	4 (0.13)	5 (0.16)	4 (0.15)	0 (0)	2 (0.11)	0 (0)	0 (0)	1 (0.10)	16 (0.10)
Others	8 (0.25)	13 (0.42)	1 (0.04)	3 (0.14)	7 (0.38)	2 (0.15)	0 (0)	1 (0.11)	35 (0.22)
β‐thalassemia	338 (10.74)	336 (10.92)	275 (10.03)	218 (10.39)	184 (9.90)	144 (10.67)	111 (11.49)	95 (10.33)	1701 (10.53)
αα/αα, β^IVS‐II‐654^/β^N^	142 (4.51)	123 (4.00)	101 (3.68)	92 (4.39)	67 (3.60)	69 (5.11)	56 (5.80)^a^	37 (4.02)	687 (4.25)
αα/αα, β^CD41‐42^/β^N^	94 (2.99)	127 (4.13)	97 (3.54)	64 (3.05)	77 (4.14)	39 (2.89)	31 (2.30)	34 (3.70)	563 (3.48)
αα/αα, β^CD17^/β^N^	37 (1.18)	40 (1.30)	26 (0.95)	19 (0.91)	17 (0.91)	7 (0.52)	7 (0.72)	10 (1.09)	163 (1.01)
αα/αα, β^‐28^/β^N^	36 (1.14)	26 (0.84)	26 (0.95)	15 (0.72)	12 (0.65)	17 (1.26)	15 (1.55)	2 (0.22)^a^	149 (0.92)
αα/αα, β^CD27‐28^/β^N^	8 (0.25)	1 (0.03)	5 (0.18)	6 (0.29)	0 (0)	3 (0.22)	2 (0.19)	4 (0.43)	29 (0.18)
αα/αα, β^E^/β^N^	1 (0.03)	0 (0)	4 (0.15)	9 (0.43)	6 (0.32)	5 (0.37)	0 (0)	2 (0.22)	27 (0.17)
αα/αα, β^CD71‐72^/β^N^	7 (0.22)	6 (0.19)	4 (0.15)	2 (0.10)	0 (0)	2 (0.15)	0 (0)	1 (0.11)	22 (0.14)
αα/αα, β^Cap^/β^N^	0 (0)	2 (0.06)	5 (0.18)	4 (0.19)	2 (0.11)	0 (0)	0 (0)	2 (0.22)	15 (0.09)
αα/αα, β^‐29^/β^N^	5 (0.16)	3 (0.10)	2 (0.07)	1 (0.05)	0 (0)	0 (0)	0 (0)	0 (0)	11 (0.07)
αα/αα, β^CD14‐15^/β^N^	2 (0.06)	0 (0)	1 (0.04)	1 (0.05)	1 (0.05)	0 (0)	0 (0)	0 (0)	5 (0.03)
Others	6 (0.19)	8 (0.26)	4 (0.15)	5 (0.24)	2 (0.11)	2 (0.15)	0 (0)	3 (0.33)	30 (0.19)

The shaded cells show that there are statistically significant differences compared with other counties.

a manifests that compared with other counties, *P* < .05.

For G6PD deficiency, there were six *G6PD* mutations and 19 genotypes were detected, including six kinds of heterozygotes, six kinds of hemizygotes, and seven kinds of homozygotes. The result of allele frequencies of *G6PD* in different counties of Meizhou area is shown in Table 4. The six *G6PD* mutations including G6PD Canton (c.1376 G → T)(45.96%), G6PD Kaiping (c.1388 G → A)(39.39%), and G6PD Gaohe (c.95 A → G)(9.09%) account for 94.44% of mutations, followed by G6PD Chinese‐5 (c.1024 C → T)(4.04%), G6PD Viangchan (c.871G → A)(1.01%), and G6PD Maewo (c.1360 C → T)(0.51%). There were higher frequency in *G6PD* c.1376 G → T allele of Wuhua (18.18%, *P* = .004) and lower frequency in *G6PD* c.1376 G → T allele of Meijiang (4.49%, *P* = .032) compared with other counties. The mutations were c.1376 G → T > c.1388 G → A > c.95 A → G in Xingning and Dabu, c.1376 G → T > c.1388 G → A > c.95 A → G in Wuhua and Meixian, c.1388 G → A > c.1376 G → T > c.1024 C → T in Meijiang, c.1388 G → A > c.1376 G → T > c.95 A → G in Fengshun, c.1388 G → A > c.1376 G → T > c.1024 C → T in Jiaoling, and c.1376 G → T > c.1388 G → A > c.1360 C → T in Pingyuan. In addition, we detected 55 patients (9.09%) with the polymorphism (c.1311 C → T).

**Table 4:** Allele frequencies of G6PD in different counties of Meizhou area

Variable	Allele number (percentage, %)								Total (n = 805)
	Xingning (n = 185)	Wuhua (n = 154)	Meixian (n = 119)	Meijiang (n = 89)	Fengshun (n = 102)	Jiaoling (n = 46)	Pingyuan (n = 52)	Dabu (n = 58)	
c.1376 G → T	19(10.27)	28 (18.18)^a^	12 (10.08)	4 (4.49)^a^	10 (9.80)	2 (4.35)	9 (17.31)	7 (12.07)	91 (11.30)
c.1388 G → A	16 (8.65)	14 (9.09)	12 (10.08)	13 (14.61)	12 (11.76)	4 (8.70)	4 (7.89)	3 (5.17)	78 (9.69)
c.95 A → G	7 (3.78)	4 (2.60)	4 (3.36)	1 (1.12)	1 (0.98)	0 (0)	0 (0)	1 (1.72)	18 (2.24)
c.1024 C → T	1 (0.54)	1 (0.65)	2 (1.68)	2 (2.25)	0 (0)	1 (2.17)	0 (0)	1 (1.72)	8 (0.99)
c.871G → A	1 (0.54)	0 (0)	1 (0.84)	0 (0)	0 (0)	0 (0)	0 (0)	0 (0)	2 (0.25)
c.1360 C → T	0 (0)	0 (0)	0 (0)	0 (0)	0 (0)	0 (0)	1 (1.92)	0 (0)	1 (0.12)
Total	44 (23.78)	47 (30.52)	31 (26.05)	20 (22.47)	23 (22.55)	7 (15.22)	14 (26.92)	12 (20.69)	198 (24.60)

The shaded cells show that there are statistically significant differences compared with other counties.

a manifests that compared with other counties, *P* < .05.

Among these cases, 130 cases were simultaneously performed on genetic testing for thalassemia and G6PD deficiency, among them, 70 cases with G6PD deficiency mutations, 27 cases carried thalassemia mutations were identified, and 13 cases with both thalassemia and G6PD deficiency mutations and 46 cases did not carry thalassemia and G6PD deficiency mutations. Due to the small number of cases, we cannot analyze the relationship of thalassemia and G6PD deficiency. Of course, further studies are required to reveal the relationship, which is one of our next major research goals.

## 4 DISCUSSION

Thalassemia is an inherited autosomal recessive disease that is mainly prevalent in Guangdong, Guangxi, and Hainan provinces. Thalassemia major places a heavy financial burden on some families.^10^, ^11^, ^12^, ^29^ Meizhou located in the northeastern part of Guangdong Province is an underdeveloped city, in which the vast majority of permanent residents are Hakka. In result of geography, culture, and customs, the proportion of intermarriage between Hakka people in Meizhou and other regions is relatively small,^24^ which consequently triggered higher genetic frequency of thalassemia. In broad terms, due to the higher prevalence and some differences from other cities of Guangdong Province, to make sure whether the eight counties of Meizhou city have some differences is necessary.

In this study, the total incidence of thalassemia in Meizhou region was 33.80%, of α‐thalassemia was 22.17%, of β‐thalassemia was 10.53%, and of α‐compound β‐thalassemia was 1.10%. Compared with previous studies, our research presented relatively higher prevalence of thalassemia because our subjects were hospitalized patients.

The statistics and analysis of molecular characteristics and morbidity of thalassemia in different regions have increasingly enriched in many studies. For example, frequency of carriers for α‐thalassemia is 15% and β‐thalassemia carriers comprise 4.8% in Guangxi Province, while 46.7% of α‐thalassemia with the main genotype of ‐‐^SEA^/αα and 39.4% of β‐thalassemia with the main genotype of CD41‐42(‐TCTT) and IVS‐II‐654(C → T) (5.8%) ranked the fifth position.^14^ The Li people in Hainan Province presented 53.45% of α‐thalassemia with chief genotype of ‐α^4.2^, ‐α^3.7^ deletion, 3.83% of β‐thalassemia with lead genotype of CD41‐42(‐TCTT) mutation, while the Han people in Hainan Province presented 12.16% of α‐thalassemia with chief genotype of ‐‐^SEA^, ‐α^3.7^ deletion, 6.11% of β‐thalassemia with the main mutation is CD41‐42(‐TCTT).^19^ As for Guangdong Province, the carrier rate of α‐thalassemia was 8.53% in which ‐‐^SEA^ and ‐α^3.7^ deletion were the main genotypes with frequencies 46.9% and 36.5%, respectively, while the carrier rate of β‐thalassemia was 2.54% in which CD41‐42(‐TCTT) (36.4%) and IVS‐II‐654(C → T) (24.8%) were the principal genotypes.^17^ There are also many differences in terms of the interior of the Guangdong Province. Shenzhen assumed 4.34% of α‐thalassemia, in which the primary genotype was ‐‐^SEA^ deletion (74.85% of mutations), 1.99% of β‐thalassemia in which CD41‐42(‐TCTT) and IVS‐II‐654(C → T) played a dominant role with frequencies 41.25% and 27.50%, respectively.^30^ Shaoguan manifested the prevalence was 10.44% of α‐thalassemia, in which ‐‐^SEA^ deletion was 3.44%, 3.97% of β‐thalassemia with chief genotype of CD41‐42(‐TCTT).^31^ The genotypes and allele frequencies of Meizhou show some differences from Guangdong. ‐‐^SEA^/αα was the most common α‐thalassemia genotype, accounted for 62.93% of α‐thalassemia. α^WS^α (CD122, CAC → CAG) accounted for 0.37% (120/32316) of α‐thalassemia and was relatively lower than the proportion of in Guangdong Province reported in the previous study. The main genotype of β‐thalassemia in Meizhou is IVS‐II‐654(C → T), whereas the counterpart in Guangdong is CD41‐42(‐TCTT); however, CD41‐42(‐TCTT) showed higher proportion in Fengshun and Meixian.

A person who is a carrier of ‐‐^SEA^ deletion is potentially at risk of begetting infants with HbH disease or the Hb Bart's hydrops fetalis syndrome, depending upon the α‐globin genotype of his/her partner, which would exhibit range from hypochromic anemia to hydrops fetalis. According to previous researches, most of regions indicated a similar feature with higher genotype of ‐‐^SEA^ deletion. Our results are consistent with those reported in most other regions. Nonetheless, the distributions and proportions in different areas regarding β‐thalassemia genotype seem to be subtly difference. Our research may not fully represent the epidemiology of thalassemia in this region related to the randomization and sample capacity of study subjects possibly as for most of our subjects were hospitalized patients.

G6PD deficiency gene carrying incidence rate was 5.41% in Guangdong Province.^32^, ^33^ The c.1388 G → A (30.4% mutations) and c.1376 G → T (25.5% mutations) were the most common *G6PD* mutations in Hakka population of Guangdong Province studied by Yu et al^34^ G6PD c.1376 G → T, c.1388 G → A, and c.95 A → G account for 78.05% mutations in Ganzhou region, Jiangxi Province,^35^ 75.3% in Han and Zhuang people in Guangxi Province,^36^ and 85.2% in Fujian Province.^37^ G6PD c.1376 G → T, c.1388 G → A, and c.95 A → G were the main type of *G6PD* gene mutations in Chinese population, which generally accounts for more than 50%. In this study, c.1376 G → T, c.1388 G → A, and c.95 A → G mutations accounted for 94.44% of the total mutation types, and the mutation distribution was consistent with the results in other places.

It is reported that the G6PD enzyme activity in patients with G6PD deficiency compound thalassemia may be higher than that of G6PD deficiency patients, due to an increase in the number of newborn erythrocytes and increase in the activity of G6PD in the chronic hemolysis of the G6PD deficiency compound thalassemia patients.^38^, ^39^, ^40^ The patients with G6PD deficiency compound thalassemia may have normal G6PD activity, so such patients may be missed in the screening of G6PD activity. We are collecting data from different types of thalassemia genotypes with *G6PD* gene mutations, and next study may reveal the correlation between thalassemia and G6PD deficiency.

The genotype distribution and frequencies of thalassemia and G6PD deficiency in Meizhou area have regional characteristics, and there are also significant differences in different counties. Local governments can formulate corresponding measures and detection projects to prevent and control thalassemia major and G6PD deficiency according to the genotype distribution and frequencies, effectively saving costs and enhancing social benefits.

## 5 CONCLUSION

In conclusion, ‐‐^SEA^, ‐α^3.7^, and ‐α^4.2^ deletion were the main mutations of α‐thalassemia, while IVS‐II‐654(C → T), CD41‐42(‐TCTT), ‐28(A → G) and CD17(A → T) mutations were the principal mutations of β‐thalassemia in Meizhou. Genetic testing for G6PD deficiency, c.1376 G → T, c.1388 G → A, and c.95 A → G were the main mutations of G6PD deficiency in this region. In addition, there were some differences of the distribution of thalassemia and *G6PD* mutations among eight counties in Meizhou.
